# The Mediating Influence of the Unified Theory of Acceptance and Use of Technology on the Relationship Between Internal Health Locus of Control and Mobile Health Adoption: Cross-sectional Study

**DOI:** 10.2196/28086

**Published:** 2021-12-29

**Authors:** Ashraf Sadat Ahadzadeh, Shin Ling Wu, Fon Sim Ong, Ruolan Deng

**Affiliations:** 1 Department of Journalism Xiamen University Malaysia Selangor Malaysia; 2 Department of Psychology School of Medical and Life Sciences Sunway University Kuala Lumpur Malaysia; 3 Malaysian Research Institute on Ageing Universiti Putra Malaysia Selangor Malaysia; 4 Department of Communication University of Vienna Vienna Austria

**Keywords:** mobile health, mHealth, internal health locus of control, performance expectancy, effort expectancy, social influence, mediation

## Abstract

**Background:**

Mobile health (mHealth) as an innovative form of information and communications technology can efficiently deliver high-quality health care by enhancing communication and health management, reducing costs, and increasing access to health services. An individual’s internal health locus of control (HLOC) is found to be associated with the behavioral intent to adopt mHealth. However, little is known about the underlying mechanism of this association.

**Objective:**

The primary objective of this study was to test the mediation influence of the Unified Theory of Acceptance and Use of Technology (UTAUT) on the relationship between internal HLOC and the behavioral intention to use mHealth.

**Methods:**

A total of 374 responses were collected from Malaysian adult users of mHealth, using convenience and snowball sampling methods. Partial least squares structural equation modeling was used to analyze the data. Data were collected for variables, including demographics, internal HLOC, and modified UTAUT constructs (ie, performance expectancy, effort expectancy, and social influence).

**Results:**

The results showed that there was no direct relationship between internal HLOC and the behavioral intention to use mHealth (β=−0.039, *P*=.32). The indirect relationship between internal HLOC and the intent to adopt mHealth was supported, indicating that the UTAUT constructs performance expectancy (β=0.104, *P*<.001), effort expectancy (β=0.056, *P*=.02), and social influence (β=0.057, *P*=.002) mediated this relationship. The results showed full mediation, with total variance explained at 47.2%.

**Conclusions:**

This study developed an integrative model, where a health-related disposition (internal HLOC), mHealth-related beliefs (performance expectancy and effort expectancy), and normative pressure (social influence) were combined to explain the underlying mechanism of the behavioral intent to adopt mHealth. The results showed that the intention to adopt mHealth is mediated by the influence of UTAUT factors, while HLOC has no direct effect on adoption intention. The findings provide insights into augmenting mHealth adoption among the public by enhancing the perceived benefits of mHealth, helping design more effective and user-friendly mHealth tools, and capitalizing on social normative influence to adopt mHealth. This study utilized the constructs of the UTAUT model to determine the intention to use mHealth. Future research should focus on other health- and technology-related theories to ascertain other possible factors influencing the behavioral intent of mHealth adoption.

## Introduction

### Background

Over the past decades, health care systems in most countries around the world have experienced tremendous changes because of the rapid advancement in information and communications technology (ICT). Mobile health (mHealth), as an innovative form of ICT, is one of the most prominent services with remarkable effects on the development of the health care system [1]. According to the Global Observatory for eHealth, mHealth is defined as “medical and public health practice supported by mobile devices, such as mobile phones, patient monitoring devices, personal digital assistants, and other wireless devices” [2]. mHealth has the potential to enhance the quality of health care systems by improving communication, efficiency, treatment adherence, and health/disease management; reducing costs; and increasing access to interventions and health services [3-6].

The popularity of mHealth programs has grown worldwide as evidenced by a Statista report in 2017 regarding the estimated number of mHealth app downloads, which has exponentially increased from 1.7 billion in 2013 to 3.7 billion in 2017 [7]. A global survey undertaken by the World Health Organization in 2011 showed that 114 countries have established mHealth initiatives, with health call centers, emergency toll-free telephone services, management of emergencies and disasters, and mobile telemedicine being the 4 most frequently reported initiatives in many countries [8]. Malaysia in Southeast Asia has started restructuring its health care policies and encouraging more start-ups to use disruptive technology to solve key medical challenges [9]. As Malaysia has a high smartphone usage (78% in 2018 [10]), mHealth adoption in the country can be a new and effective approach to empower people in health management and transform the attitude toward health care from reactive to proactive [11].

The importance and implications of mHealth have inspired researchers to investigate the factors in the adoption of mHealth. A cluster of studies viewed mHealth as a perceived technology–driven behavior and attempted to find the correlates of such behavior using technology adoption theories [12-14]. Another stream of studies attempted to explain mHealth from health-related perspectives and examined health factors as predictors of mHealth adoption [15]. While the former has been widely researched, the latter has received less attention.

Among health-related factors, the belief that health events are caused by one’s own actions is one of the major predictors of health behaviors such as greater engagement in health/disease management, healthier lifestyle, and better physical and mental quality of life [16,17]. Those who believe that they have an active role in one’s own health, also known as internal health locus of control (HLOC), are more likely to take responsibility toward their health and engage in health behaviors [18]. Given that individuals’ health-related dispositional factors are significant antecedents of health behaviors [19], including the use of health technologies [20-22], very limited studies have examined the relationship between internal HLOC and the intent to adopt mHealth [23].

Although the direct relationship between internal HLOC and behavioral intention to use mHealth provides a noteworthy tenet to knowledge, little is known regarding the underlying mechanism of this relationship. Focusing on mediating factors, which facilitate the relationship between internal HLOC and the intent to use mHealth, can provide a better insight to Malaysian health policy makers and health care professionals for embedding mHealth use in daily life and promoting mHealth functions (such as health/disease information seeking, communicating for health-related purposes, and downloading and using any health-related apps) for health management and more importantly active participation in disease prevention, thus reducing the need for health care services and consequently the toll on the Malaysian health care system.

Therefore, this study aims to contribute to the literature by introducing 3 constructs of the Unified Theory of Acceptance and Use of Technology (UTAUT) (ie, performance expectancy, effort expectancy, and social influence) as mediators between internal HLOC and the intention to use mHealth, which, to the best of our knowledge, has not been examined thus far. The UTAUT is one of the most widely accepted technology adoption theories with a wide applicability and a high explanatory power to predict the intent to adopt technology [24]. By integrating the UTAUT constructs as mediators in the association between internal HLOC and the intention to use mHealth, a mediation model has been developed to explain the mechanism that underlies the relationship between internal HLOC and behavioral intent to adopt mHealth. Therefore, the objectives of this study are to investigate (1) the relationship between internal HLOC and behavioral intent to adopt mHealth; (2) the relationship between the constructs of the UTAUT (ie, performance expectancy, effort expectancy, and social influence) and the intention to adopt mHealth; (3) the relationship between internal HLOC and the constructs of the UTAUT; and (4) the mediation effects of performance expectancy, effort expectancy, and social influence in the relationship between internal HLOC and the intent to adopt mHealth.

### Literature

#### HLOC and Technology-Related Behaviors

Locus of control (LOC) is a psychological construct that is derived from the social learning theory of personality [25]. Wallston et al [18] developed a multidimensional HLOC scale, which denotes how much individuals believe they are in control of their current and future health. HLOC can be measured as an internal or external HLOC [18]. Internal HLOC posits an active role in one’s own health and taking responsibility toward health, whereas external HLOC is divided into 2 parts, with one referring to powerful others and the other referring to chance, luck, or the influence of religion [18].

In general, higher levels of internal HLOC are more likely to drive healthy behaviors and more successful changes in health behaviors and preventive health behaviors, whereas higher levels of external HLOC are not. Stronger internal HLOC orientations were found to be related to greater engagement in health-enhancing behaviors (such as exercise and diet) [16,26,27]; better mental and physical quality of life [16]; lower rates of smoking and excessive drinking [28,29]; better medication adherence [30]; lower levels of stress, depression, and anxiety [16,31]; longer survival time after surgery [32]; and better health rehabilitation and care [33,34]. Moreover, individuals with internal HLOC tend to actively use coping strategies that focus on solving problems [17] and show greater beliefs in the ability to control the risk of disease [35,36]. Conversely, those with high chance HLOC believe that there is nothing much they can do to influence their health outcomes, and consequently, they are less likely to engage in health behaviors [37]. Additionally, those with higher external LOC are more likely to report higher levels of stress [38] and have worse mental health, as they use more emotion-focused strategies [17].

Research into technology adoption has relied on LOC as a construct that explains adoption behavior. Empirical studies provided support for the association between LOC and higher propensity of adopting technology in an array of technologies where differences in internal and external LOC tend to differentiate the behaviors between these 2 groups [39-42]. Individuals with internal LOC tend to use technology in a goal-directed manner; they are more likely to adopt problem-solving stances to change the environment compared with externals [43]. Internals are more likely to have higher utilization of the technology [44,45]. They are early adopters of technology, are more satisfied with their skills to use technology, and are more comfortable with technology [43], and they perceive difficulties in using technology as associated with their own lack of abilities [46]. Given the rising technology use across all demographic groups, an emerging cluster of scholarly works has been devoted to internal HLOC to predict the intention to adopt mHealth [23].

#### The Mediating Effect of UTAUT Constructs

Limited research on the relationship between internal HLOC and behavioral intent to adopt mHealth calls for further investigation into the possibility of other variables that could underlie this relationship. Therefore, this study intends to suggest the mediating effect of UTAUT constructs to test the indirect relationship between internal HLOC and the intention to use mHealth. Venkatesh et al developed a unified model that has an overall comprehensive explanatory power to conceptualize and predict acceptance behavior, known as the UTAUT [47]. Numerous UTAUT studies have verified that 3 core constructs (ie, performance expectancy, effort expectancy, and social influence) can affect the intention to adopt technology [48-54]. Performance expectancy refers to “the degree to which an individual believes that using the system will help him or her to attain gains in job performance” [47]. Effort expectancy is defined “as the degree of ease associated with the use of the system” [47]. Social influence is defined “as the degree to which an individual perceives that important others believe he or she should use the new system” [47]. Performance expectancy, effort expectancy, and social influence have direct effects on behavioral intention, which in turn predicts use behavior [47].

Since its emergence, the UTAUT has been empirically tested across domains [53-55], including eHealth and mHealth [56,57]. Past studies have shown that performance expectancy, effort expectancy, and social influence are significantly associated with the intention to use eHealth and mHealth in elderly people and citizens dealing with a health problem [12,52]. Application of the UTAUT to examine the intention to use eHealth and mHealth among clinicians and health care professionals has also been endorsed in a handful of studies [14,56]. It is understood that the target population in most past studies was mainly older adults, people with a health problem, and health professionals. This study attempts to investigate the associations between the 3 constructs of UTAUT (ie, performance expectancy, effort expectancy and social influence, and intention to use mHealth) among Malaysians aged 18 years or above. Evidence showed that although most Malaysians have limited knowledge about mHealth, they reported having a positive attitude toward mHealth [58]. Likewise, a favorable affective feeling was also reported toward telemonitoring systems by patients [59], telemedicine technology by health providers [60], e-counseling by counselors [61], and internet usage for health-related purposes (such as health information seeking and communication) by women [20].

Individuals who score high in internal LOC, also known as internals, cherish innovative ideas [62], tend to be early adaptors of innovative products [44,63], and perceive technology as useful [41,42,46], thus showing a stronger tendency toward technology adoption [64]. They also master their learning environment using more proactive approaches and believe that using technology is free of effort [65]. Internals are characterized by high self-efficacy, which is the impetus to overcome difficulties in using technologies [55]. They are more likely to demonstrate a positive attitude toward computers [65,66], and greater confidence and lower levels of anxiety in using computers [67]. Internal LOC has also been empirically found to be associated with social influence [68,69]. Individuals with higher internal LOC tend to be less likely to be influenced by others [70].

In light of the above literature, we would expect internal HLOC to predict UTAUT constructs, which may, in turn, predict behavioral intent to adopt mHealth. In other words, instead of a direct relationship between HLOC and mHealth, we would expect an indirect relationship that could provide an underlying mechanism to explain how those high on internal HLOC are disposed toward mHealth use. Individuals who tend to assign the cause of health outcomes to their internal characteristics rather than to outside forces are more likely to perceive that mHealth is useful and easy to use. However, they may not use mHealth because of social influences as they do not believe that external forces, such as others, can motivate them to use mHealth. Because of their belief in mHealth usefulness and ease of use, they may have an intention to adopt mHealth. However, their resistant to social influence may hinder them from adopting mHealth. Hypotheses and justifications for the hypotheses are presented in Textbox 1.

**Hypotheses and justifications for the hypotheses.**

**Hypothesis 1: Internal health locus of control (HLOC) has a positive relationship with the intention to adopt mobile health (mHealth).**

*Justifications for the hypothesis*

- It was found that there is a relationship between locus of control (LOC) and technology adoption in developing agriculture [39], online games [40], and online learning [41,42].
- Individuals with oropharyngeal head and neck cancer with a high propensity for an internal HLOC orientation showed their support toward telepractice models of care telerehabilitation [22].
- A cross-sectional study revealed that the amount of control college students believed they had over their health predicted willingness to use health apps and online health trackers [23].

**Hypothesis 2: Performance expectancy has a positive relationship with the intention to adopt mHealth.**

**Hypothesis 3: Effort expectancy has a positive relationship with the intention to adopt mHealth.**

**Hypothesis 4: Social influence has a positive relationship with the intention to adopt mHealth.**

*Justifications for the hypotheses*

- In a study to examine the intention of elderly people aged 57 to 77 years to use eHealth apps, expected performance and effort were highly related to the intention to use eHealth while social influence was not [52].
- A study revealed that Unified Theory of Acceptance and Use of Technology factors, namely effort expectancy, expectancy performance, and social influence, were significant determinants of the intention for mHealth adoption behavior in citizens like diabetic patients who were taking traditional health care services repeatedly from any medical hospital for diabetes, blood pressure, and cholesterol monitoring in the United States, Canada, and Bangladesh [12].
- A study on the intention to use a mobile electronic health record (MEHR) system in a sample of health care professionals (doctors and nurses) showed that the intention to use the MEHR system was indirectly influenced by effort expectancy and performance expectancy through attitudes toward the MEHR system, while social influence was found to be directly associated with the intention to utilize the MEHR system [14].
- Venugopal et al [56] examined clinical staff’s perspectives on the usage of telemedicine and electronic health records in hospitals and found that performance expectancy, effort expectancy, and social influence have a significant impact on intention, which in turn impacts the usage behavior of electronic health records and telemedicine.

**Hypothesis 5: Internal HLOC has a positive relationship with performance expectancy.**

*Justifications for the hypothesis*

- Individuals with high internal LOC are more likely to seek new information when the information is personally relevant, and obtain valuable knowledge and skills to enhance their performance [65,71].
- Internals commonly display a favorable attitude toward technology [66].

**Hypothesis 6: Internal HLOC has a positive relationship with effort expectancy.**

*Justifications for the hypothesis*

- Individuals with internal LOC attributed perceived difficulty toward technology to their own abilities and attempted to use technology more effectively [46].
- Internals have more experience in using technologies and find technology, such as e-learning, easy to use [41,42,72,73].

**Hypothesis 7: Internal HLOC has a negative relationship with social influence.**

*Justifications for the hypothesis*

- Individuals with higher internal LOC are resistant to social influence as they feel they have more self-control and self-reinforcement over their life and things that happened to them [71].
- They are not easily persuaded and do not conform to others’ influence [68].

**Hypothesis 8: Performance expectancy mediates the positive relationship between internal HLOC and the intention to adopt mHealth.**

**Hypothesis 9: Effort expectancy mediates the positive relationship between internal HLOC and the intention to adopt mHealth.**

**Hypothesis 10: Social influence mediates the positive relationship between internal HLOC and the intention to adopt mHealth.**

*Justifications for the hypotheses*

- Performance expectancy and effort expectancy were found to be positive and significant mediators among website design, customer service, and customer’s intention to adopt internet banking [74].
- Performance expectancy and effort expectancy were found to be linked to user adoption in context awareness and Alipay, a third-party mobile and online payment platform [75].
- Fong et al [55] explored the mediating role of effort expectancy and perceived risk in the relationship between internal LOC and the intention to reuse mobile apps for making hotel reservations.

## Methods

### Participant Profiles

Among 374 participants in this study, there were 145 males and 229 females. The participant age ranged from 18 to 68 years (mean 28.01 years, SD 11.10). Almost 45% (166/374, 44.4%) of the participants were Chinese, and 40.7% (152/374) were Malays. In terms of health status, 47.4% (177/374) of the participants perceived their health status as good, 27.5% (103/374) perceived it as fair, and 18.2% (68/374) perceived it as very good. Participants were also asked whether they had an ongoing or a serious health problem that included heart disease, arthritis, or a mental health condition requiring frequent medical care, such as regular visits to doctors or daily medications. The majority (315/374, 84.3%) of the participants indicated that they did not have any ongoing or serious health problem, while 12.0% (45/374) reported that they did not know of any serious health problems. A small percentage (14/374, 3.7%) of participants had an ongoing disease or serious health problem. Lastly, regarding mobile phone usage experience, 40.4% (151/374) of the participants had 8 to 10 years of experience, 39.0% (146/374) had 4 to 7 years of experience, 15.0% (56/374) had more than 10 years of experience, and 5.6% (21/374) had 1 to 3 years of experience. Table 1 shows the demographic profile of the respondents.

**Table 1:** Demographic profile of the respondents.

Background variable			Value (N=374), n (%)
**Gender**			
	Male	145 (38.8)	
	Female	229 (61.2)	
**Ethnicity**			
	Malay	152 (40.7)	
	Chinese	166 (44.4)	
	Indian	47 (12.5)	
	Others	9 (2.4)	
**Perceived health status**			
	Do not know	3 (0.8)	
	Poor	9 (2.4)	
	Fair	103 (27.5)	
	Good	177 (47.4)	
	Very good	68 (18.2)	
	Excellent	14 (3.7)	
**Disease**			
	Yes	14 (3.7)	
	No	315 (84.3)	
	Do not know	45 (12.0)	
**Mobile phone usage experience**			
	1-3 years	21 (5.6)	
	4-7 years	146 (39.0)	
	8-10 years	151 (40.4)	
	>10 years	56 (15.0)	

### Research Design and Procedure

This study used a questionnaire-based cross-sectional design to collect the required data. A total of 400 questionnaires were distributed to Malaysian adults residing in Kuala Lumpur, Malaysia. The subjects for this study were drawn from mHealth users. A screening self-report question was included in the survey to identify mHealth users. Participants were asked if they have ever used their smartphones for any health-related purposes, such as seeking health- and disease-related information online, texting messages for health-related purposes (such as reminders/alerts for appointments, taking medications, and consultations), and downloading and using any health-related apps (such as fitness apps, and apps for health tracking and medication tracking). Participants who reported having used their smartphones at least for one of these purposes were included in the analysis.

The questionnaire comprised an informed consent form, demographic profiles, and questions related to internal HLOC and the modified UTAUT constructs for mHealth use. Data were collected using convenience and snowball sampling methods. A research assistant was recruited for data collection. Participation was voluntary where confidentiality was ensured, and respondent consent was obtained before commencing the survey. Participants were given an absolute right of withdrawal at any time and without giving any reason. The protocol of the study (including the research procedure, the rights and safety of the participants, and the method of data collection) was approved by the review board of Xiamen University Malaysia to ensure compliance with research ethics. The approval number is REC-1911.01.

The following 2 rules of thumb are used for choosing the sample size when partial least square is to be used for model analysis: (1) “10 times the scale with the largest number of formative (ie, causal) indicators (note that scales for constructs designated with reflective indicators can be ignored),” and (2) “10 times the largest number of structural paths directed at a particular construct in the structural model.” [76]. This study did not have any formative indicator. Therefore, the first rule of thumb was not applicable for this study. According to the former rule of thumb, the minimum sample size for this study is 70, as the largest number of structural paths in the research model is 7, which is related to the behavioral intent to adopt mHealth. Further, recent developments suggest that researchers should determine sample size through power analysis [77,78]. Power analysis determines the minimum sample size by taking into account the part of a model with the largest number of predictors [79]. Therefore, we used G*Power to determine the required sample size. Based on G*Power, the adequacy of sample size was computed using the statistical tests recommended. For a mediation model, the input information was as follows: test family: “F tests,” statistical test: “linear multiple regression: fixed model, R2 deviation from zero,” type of power analysis: “a priori: compute required sample size–given α, power, and effect size,” with effect size=0.15, α=.05, power=0.90, and number of predictors=4. G*Power showed that the minimum sample size required for the mediation model is 108, with an actual power of 0.90. Therefore, the number of responses collected for this study was sufficient for analysis.

### Measurements

#### Internal HLOC

In measuring internal HLOC, 6 items were adopted from the Multidimensional Health Locus of Control scale developed by Wallston et al [18]. Respondents were asked to rate items on a 5-point Likert scale from 1 (strongly disagree) to 5 (strongly agree).

#### Performance Expectancy, Effort Expectancy, Social Influence, and Intention to Use mHealth

Items to measure performance expectancy, effort expectancy, social influence, and intention to use mHealth were directly extracted from the original UTAUT model [24,47] and modified to make them consistent with mHealth use behavior. Participants were requested to rate the items on a 5-point Likert scale ranging from 1 (strongly disagree) to 5 (strongly agree).

Questions related to internal HLOC and the modified UTAUT constructs for mHealth use are included in Multimedia Appendix 1.

## Results

### Data Analysis

In this study, partial least squares structural equation modeling (PLS-SEM) was used to examine the proposed conceptual framework using SmartPLS software. By using PLS-SEM, the direct and indirect effects of multiple independent and dependent variables can be tested simultaneously, which provides greater statistical power. PLS-SEM is also able to accommodate a study with a small sample size despite the complexity of the models [79]. After excluding incomplete questionnaires, 374 responses were included in the analysis. The results consisted of a 2-stage analytical procedure, which involved a measurement model and structural model, to validate the model and test the hypotheses developed for the purpose of this study.

### Measurement Model

The first step in the analysis concerning the measurement model was to examine the factor loading. In this study, the factor loadings of the items varied from 0.632 to 0.945 (Table 2). All items were retained as the factor loadings were above the recommended value of 0.6 [80]. Second, composite reliability, which measures the internal consistency of the constructs, was used to measure the reliability of the variables. According to Hair et al [79], the minimum value of composite reliability is 0.7. Table 2 shows that all the constructs yielded good composite reliability ranging from 0.873 to 0.954. Third, convergent validity was assessed using average variance extracted (AVE). Hair et al [79] mentioned that the minimum acceptable value for AVE is 0.5 or higher, which indicated that 50% or more of the items were explained by the construct. In this study, all the constructs exceeded the acceptable value, which indicated that all the constructs obtained good convergent validity (Table 2).

**Table 2:** Assessment results of the measurement model.

Constructs and items			Loading		CR^a^	AVE^b^
**Internal health locus of control**					0.873	0.535
	IHLC1	0.714				
	IHLC2	0.662				
	IHLC3	0.799				
	IHLC4	0.747				
	IHLC5	0.817				
	IHLC6	0.632				
**Performance expectancy**					0.915	0.783
	PE1	0.853				
	PE2	0.920				
	PE3	0.880				
**Effort expectancy**					0.937	0.789
	EE1	0.875				
	EE2	0.890				
	EE3	0.926				
	EE4	0.861				
**Social influence**					0.954	0.874
	SI1	0.919				
	SI2	0.945				
	SI3	0.940				
**Behavioral intention**					0.891	0.732
	BI1	0.785				
	BI2	0.898				
	BI3	0.880				

^a^CR: composite reliability.

^b^AVE: average variance extracted.

Lastly, discriminant validity was determined using the heterotrait-monotrait (HTMT) ratio of correlation as recommended by Henseler et al [81] because it is more suitable for discriminant validity assessment compared with the Fornell-Larcker criterion and cross-loading assessment. In order to achieve discriminant validity, Henseler et al suggested that a threshold value of 0.90 or below is required [81]. As shown in Table 3, all the study variables met the criterion to establish discriminant validity where the HTMT values were below 0.90.

**Table 3:** Discriminant validity using the heterotrait-monotrait (HTMT) ratio.

Construct	Internal health locus of control	Performance expectancy	Effort expectancy	Social influence	Behavioral intention
Internal health locus of control	N/A^a^	0.390	0.373	0.212	0.213
Performance expectancy	0.390	N/A	0.886	0.687	0.745
Effort expectancy	0.373	0.886	N/A	0.636	0.678
Social influence	0.212	0.687	0.636	N/A	0.682
Behavioral intention	0.213	0.745	0.678	0.682	N/A

^a^N/A: not applicable.

### Structural Model

Multicollinearity is assessed using the variance inflation factor (VIF). In this study, all VIF values were below 5, which indicated no violation of the multicollinearity assumption. The structural model was assessed using *R*^2^, beta, and *t* value, which were obtained via a 5000 resampling of the bootstrapping procedure. The direct, total indirect, and specific indirect effects are shown in Table 4.

**Table 4:** Direct, total indirect, and specific indirect effects.

Path				*R* ^2^		Beta		*t* value		*P* value
**Direct effects**										
	**Behavioral intention**		0.472							
		Internal health locus of control → behavioral intention			−0.039		0.999		.32	
		Performance expectancy → behavioral intention			0.316		4.859		<.001	
		Effort expectancy → behavioral intention			0.169		2.672		.008	
		Social influence → behavioral intention			0.307		5.715		<.001	
	**Performance expectancy**		0.109							
		Internal health locus of control → performance expectancy			0.330		6.522		<.001	
	**Effort expectancy**		0.109							
		Internal health locus of control → effort expectancy			0.329		7.020		<.001	
	**Social influence**		0.034							
		Internal health locus of control → social influence			0.186		3.621		<.001	
**Total indirect effects**										
	**Behavior**		0.472							
		Internal health locus of control → behavior through performance expectancy, effort expectancy, and social influence			0.217		5.554		<.001	
**Specific indirect effects**										
	Internal health locus of control → performance expectancy → behavioral intention				0.104		3.813		<.001	
	Internal health locus of control → effort expectancy → behavioral intention				0.056		2.389		.02	
	Internal health locus of control → social influence → behavioral intention				0.057		3.123		.002	

**Figure 1:**
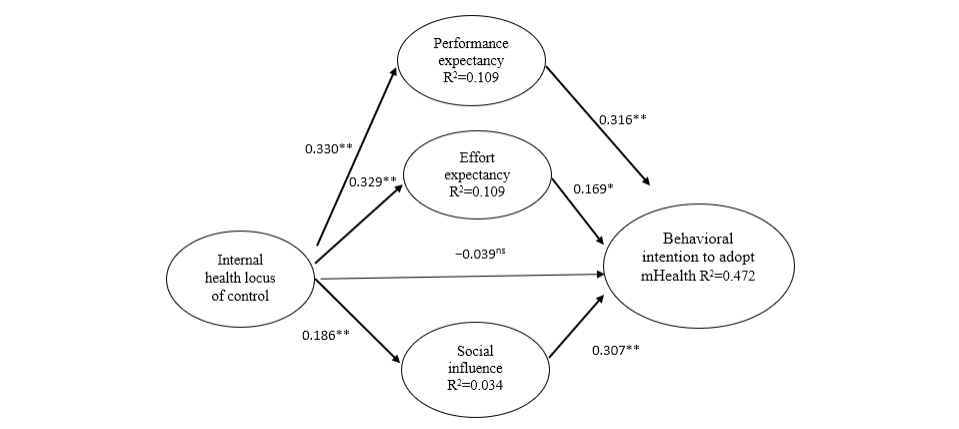
Path coefficients of the structural research model. mHealth: mobile health; ns: not significant. *P<.01, **P<.001.

Based on the structural model, hypothesis 1 was not supported because internal HLOC (β=−0.039, *P*=.32) was not significantly related to the intention to use mHealth. Performance expectancy (β=0.316, *P*<.001), effort expectancy (β=0.169, *P*=.008), and social influence (β=0.307, *P*<.001) had a significant and positive relationship with the intention to use mHealth, which supported hypotheses 2, 3, and 4 (Figure 1). Hypotheses 5, 6, and 7 were also supported where results showed that internal HLOC had a significant positive relationship with performance expectancy (β=0.330, *P*<.001), effort expectancy (β=0.329, *P*<.001), and social influence (β=0.186, *P*<.001).

Overall, the results supported the model of this study where the total indirect effect was significant (β=0.217, *P*<.001). All the study variables explained 47.2% of the variance in the intention to use mHealth. Three specific indirect paths were generated to test hypotheses 8 to 10. Hypothesis 8 was developed to test the mediating effect of performance expectancy on the relationship between internal HLOC and the intention to adopt mHealth. The results showed that the direct effect of internal HLOC on performance expectancy and the direct effect of performance expectancy on the intention to use mHealth were significant, while the direct effect of internal HLOC on the intention to use mHealth was not significant. This indicated that performance expectancy fully mediated the relationship between internal HLOC and the intention to use mHealth with a significant indirect effect (β=0.104, *P*<.001), supporting hypothesis 8 (Figure 1).

For hypothesis 9, effort expectancy as the mediator for the relationship between internal HLOC and the intention to adopt mHealth was tested. The direct effect from internal HLOC to effort expectancy and the direct effect from effort expectancy to the intention to use mHealth were significant, but the direct effect from internal HLOC to the intention of adopting mHealth was not significant. This indicated that effort expectancy fully mediated the relationship between internal HLOC and the intention to use mHealth (β=0.056, *P*=.02). Thus, hypothesis 9 was supported (Figure 1).

In Figure 1, the results showed support for hypothesis 10, where social influence significantly mediated the relationship between internal HLOC and the intention to use mHealth. The significant indirect effect (β=0.057, *P*=.002) revealed that internal HLOC impacted social influence, and social influence in turn affected the intention to use mHealth. Based on the findings of the mediation tests, it can be seen that the mediating effect of performance expectancy on the relationship between internal HLOC and the intention to use mHealth was the strongest, followed by social influence and effort expectancy.

## Discussion

Previous studies have demonstrated the role of LOC in the tendency to use technology [39-42,55]. Since research on mHealth behavior is limited and little is known about how LOC could influence the intention to adopt mHealth technology, this study aimed to find out the factors that could explain users’ behavioral intent in mHealth technology. Drawing upon 3 constructs of the UTAUT, this study attempted to provide insights on how internal HLOC delineates the intention to adopt mHealth.

In this study, internal HLOC was not found to be significantly related to the intention to use mHealth (hypothesis 1). This result is inconsistent with previous research providing evidence that individuals with internal LOC beliefs tend to utilize health apps [23]. Some studies showed a negligible relationship between HLOC and health behaviors [22,82,83], thus leading to inconsistent findings about the significance of HLOC in driving health behaviors. Calnan [84] suggested that health behaviors may not be associated with beliefs regarding control of health but rather with concerns over risky health behaviors. The absence of a relationship between internal HLOC and behavioral intent to adopt mHealth could also be attributed to potential confounding factors, such as health literacy, that could influence HLOC, which in turn affects the intention to use mHealth. Health literacy was found to be an effective factor in predicting internal orientations [85]. Individuals with higher levels of health literacy are more likely to report higher scores of internal HLOC than those with lower levels of health literacy because their capacities to obtain, process, and understand health information and services in order to make appropriate health decisions are positively associated with their belief that they have control over their health and health behaviors [86]. Another possible confounding factor that may affect internal HLOC is the participants’ economic status. Research has found that individuals with lower socioeconomic status engaged in fewer health-promoting behaviors and had different expectations about their ability to influence their own health [87]. People living in economically deprived circumstances may, as a result of their experiences, also learn that they have less control over their own lives and health status [88].

The UTAUT constructs, namely performance expectancy, effort expectancy, and social influence, were found to be significant predictors, with a positive relationship for the intention to use mHealth (hypotheses 2, 3, and 4), lending support to past studies that consistently showed the association between UTAUT determinants and eHealth and mHealth adoption [12,52,56,57]. All these findings unfailingly corroborate the UTAUT model, where if users perceive mHealth as useful, easy to use, and accepted as well as suggested by important others, they will be more likely to adopt the technology. Among these 3 constructs, performance expectancy and effort expectancy have equal contributory impact on mHealth adoption, followed by social influence. These results are consistent with the findings in the study by Tavares and Oliveira [57]. They found that performance expectancy and effort expectancy predict the same variance in the use of online services such as electronic health record portals. It suggests that usefulness and ease of use are equally central for users when evaluating mHealth services [57]. Besides, the power of important others should not be neglected in technology adoption behavior. Research has found that social influence is an essential source of motivation to utilize hospital electronic information management systems [56]. In contrast, normative pressure was found to be an insignificant force to patient portal use behavior among the elderly [89]. These contradicting results can lead us to an inference that social influence is more likely to be a driving force for technology adoption behavior among young individuals rather than older people because “older people do not follow the bandwagon effect” [90].

To further explore whether internal HLOC can be suitably applied in the UTAUT model, the relationships between internal HLOC and 3 constructs of the UTAUT (ie, performance expectancy, effort expectancy, and social influence) in the mHealth context were postulated in this study (hypotheses 5, 6, and 7). The results showed that internal HLOC had a significant positive effect on performance expectancy, effort expectancy, and social influence in mHealth use, which suggests that the more internal the users are, the higher the perceived usefulness and ease of use they will have and the more likely they will conform to important others. These results are in line with the findings of previous studies that showed the significant influence of LOC on perceived usefulness and ease of use for mobile learning adoption [40,41]. In addition, Fong et al [55] found that internals are characterized by high self-efficacy, a trait that helps individuals to overcome difficulties. Hence, they perceive higher ease of use for new technology products compared with externals [72]. As opposed to the negative association hypothesized between internal HLOC and social influence, this study found a positive relationship between these 2 constructs. This could be due to the individual differences in tendencies to conform for informational reasons but not for normative reasons [73].

In testing the mediating role of performance expectancy, effort expectancy, and social influence, this study found that these 3 constructs fully mediated the relationship between internal HLOC and the intention to use mHealth, supporting hypotheses 8, 9, and 10. Internal HLOC was positively related to the intention to adopt mHealth through performance expectancy. Effort expectancy had a mediating effect on the relationship between internal HLOC and the intention to use mHealth (hypothesis 9), similar to the results found in previous research [55]. Individuals with internal HLOC are more likely to overcome difficulties in using new technology products. They prefer to adopt mHealth because it is easy to use. Moreover, social influence was found to significantly mediate the relationship between internal HLOC and the intention to use mHealth (hypothesis 10). Internals use mHealth partly because of the perception that important others may suggest to employ mHealth. Individuals’ beliefs in their own efforts and abilities to control their health drive perceptions toward mHealth, which in turn contribute to mHealth adoption. UTAUT dimensions are central to our understanding of the association between internal HLOC and the intention to adopt mHealth. The mediation results of this study are consistent with the findings in the study by Ahadzadeh et al [20]. The authors found the centrality of perceived usefulness and perceived ease of use in the relationship between health factors and internet use for health-related purposes. A strong mediating effect of health app use efficacy was also identified in the effect of health information orientation and eHealth literacy on health app use [91].

The findings of this study have several implications. Theoretically, this study has contributed to mHealth literature by investigating the direct and indirect relationships between internal HLOC and the intention to use mHealth. The indirect relationship provided a more multifaceted understanding of mHealth adoption behavior, where both health and technology come into play in the adoption decision process. Moreover, the results attested the robustness of the UTAUT in mHealth adoption. To the best of our knowledge, this study is the first attempt to examine the indirect effect of internal HLOC on the behavioral intent of mHealth. The results of the effect of UTAUT dimensions on the intention to adopt mHealth have significant implications for health providers seeking methods to enhance mHealth engagement behavior. They can leverage cognitive and normative factors related to technology (ie, performance expectancy, effort expectancy, and social influence) to increase individuals’ preferences to use mHealth for health purposes.

The limitations of this study can be attributed to the urban sample concentrated in the most developed part of Malaysia. A more representative sample should be considered in future studies. Nonprobability sampling methods (ie, convenience and snowball) employed for the sample selection and the unbalanced gender makeup of the sample can jeopardize the generalizability of the results. The cross-sectional design used in this research does not provide definite information about cause and effect relationships. Moreover, social desirability bias can be a problem with self-report measurements used in this study. The framework proposed in this study predicted 42.7% of the variance in mHealth, while a more extended model encompassing more cognitive factors can augment the prediction power of the model. Given the absence of a significant association between internal HLOC and the intent to use mHealth in this research, replication studies are suggested, which can include a broader framework of multiple health-related factors (such as perceived health susceptibility, perceived health severity, perceived health status, and health consciousness) and personality factors in order to advance the frontier of knowledge regarding HLOC and technology adoption, since technology will become even more important in the future with “Industrial Revolution 4.0.” Moreover, incorporating perceived health risk factors along with HLOC will enable researchers to determine whether mHealth is a proactive/preventive health behavior, a reactive behavior, or both.

## Appendix

Multimedia Appendix 1 Questions related to internal health locus of control and the modified Unified Theory of Acceptance and Use of Technology constructs.

## Abbreviations

AVE: average variance extracted
HLOC: health locus of control
HTMT: heterotrait-monotrait
ICT: information and communications technology
LOC: locus of control
mHealth: mobile health
PLS-SEM: partial least squares structural equation modeling
UTAUT: Unified Theory of Acceptance and Use of Technology
VIF: variance inflation factor
