# Supplementary material for: The Mediating Influence of the Unified Theory of Acceptance and Use of Technology on the Relationship Between Internal Health Locus of Control and Mobile Health Adoption: Cross-sectional Study
Source: J Med Internet Res. 2021 Dec 29;23(12):e28086. doi: 10.2196/28086 (PMC8756342; doi:10.2196/28086)
Supplement: Multimedia Appendix 1 [file jmir_v23i12e28086_app1.docx]

**Multimedia Appendix 1.** Questions related to internal health locus of control and the modified Unified Theory of Acceptance and Use of Technology constructs.

| **Variables** | **Items** | **Source** |
| --- | --- | --- |
| **Performance Expectancy** | I would find mHealth services useful in my daily life | [24,47] |
|  | Using mHealth services helps me accomplish things more quickly |  |
|  | Using mHealth services increases my productivity |  |
| **Effort Expectancy** | Learning how to use mHealth service is easy for me |  |
|  | My interaction with mHealth services is clear and understandable |  |
|  | I would find mHealth services easy to use |  |
|  | It is easy for me to become skillful at using mHealth services |  |
| **Social Influence** | People who are important to me think that I should use mHealth service |  |
|  | People who influence my behaviour think that I should use mHealth service |  |
|  | People whose opinions that I value prefer that I use mHealth service. |  |
| **Behavioral Intention** | I intend to use mHealth service in the future |  |
|  | I will always try to use mHealth service in my daily life |  |
|  | I plan to use mHealth service frequently |  |
| **Internal Locus of**  **Control** | I can pretty much stay healthy by taking good care of myself. . | [18] |
|  | If I get sick, I have the power to make myself well again. |  |
|  | I am directly responsible for my health. |  |
|  | When I feel ill, I know it is because I have not been taking care of myself properly. |  |
|  | My physical well-being depends on how well I take care of myself. |  |
|  | Whatever goes wrong with my health is my own fault. |  |
